# Review Article: Diagnostic Paradigm Shift in Spine Surgery

**DOI:** 10.3390/diagnostics15050594

**Published:** 2025-02-28

**Authors:** Aras Efe Levent, Masato Tanaka, Chetan Kumawat, Christian Heng, Salamalikis Nikolaos, Kajetan Latka, Akiyoshi Miyamoto, Tadashi Komatsubara, Shinya Arataki, Yoshiaki Oda, Kensuke Shinohara, Koji Uotani

**Affiliations:** 1 Department of Orthopedic Surgery, Okayama Rosai Hospital, 1-10-25 Chikkomidorimachi, Minami Ward Okayama, Okayama 702-8055, Japan; efe_aras2002@yahoo.com (A.E.L.); dr.ckumawat@gmail.com (C.K.); heng.christian@gmail.com (C.H.); salamalikis.orthopedics@gmail.com (S.N.); kajetan.latka@uni.opole.pl (K.L.); akkun@kzd.biglobe.ne.jp (A.M.); t.komatsubara1982@gmail.com (T.K.); araoyc@gmail.com (S.A.); 2 Department of Orthopedic Surgery, Sir Ganga Ram Hospital, Rajinder Nagar, New Delhi 110060, India; 3 Department of Orthopedic Surgery, Okayama University Hospital, Okayama 7000-8558, Japan; odaaaaaaamn@yahoo.co.jp (Y.O.); joker1011ks@yahoo.co.jp (K.S.); coji.uo@gmail.com (K.U.)

**Keywords:** diagnosis, spine surgery, innovative technique, MRI, myelography

## Abstract

Meticulous clinical examination is essential for spinal disorders to utilize the diagnostic methods and technologies that strongly support physicians and enhance clinical practice. A significant change in the approach to diagnosing spinal disorders has occurred in the last three decades, which has enhanced a more nuanced understanding of spine pathology. Traditional radiographic methods such as conventional and functional X-rays and CT scans are still the first line in the diagnosis of spinal disorders due to their low cost and accessibility. As more advanced imaging technologies become increasingly available worldwide, there is a constantly increasing trend in MRI scans for detecting spinal pathologies and making treatment decisions. Not only do MRI scans have superior diagnostic capabilities, but they also assist surgeons in performing meticulous preoperative planning, making them currently the most widely used diagnostic tool for spinal disorders. Positron Emission Tomography (PET) can help detect inflammatory lesions, infections, and tumors. Other advanced diagnostic tools such as CT/MRI fusion image, Functional Magnetic Resonance Imaging (fMRI), Upright and Kinetic MRI, magnetic resonance spectroscopy (MRS), diffusion-weighted imaging (DWI), and diffusion tensor imaging (DTI) could play an important role when it comes to detecting more special pathologies. However, some technical difficulties in the daily praxis and their high costs act as obstacles to their further spread. Integrating artificial intelligence and advancements in data analytics and virtual reality promises to enhance spinal procedures’ precision, safety, and efficacy. As these technologies continue to develop, they will play a critical role in transforming spinal surgery. This paradigm shift emphasizes the importance of continuous innovation and adaptability in improving the diagnosis and treatment of spinal disorders.

## 1. Introduction

Spine surgery has evolved remarkably, shaped by advances in medical knowledge, technology, and surgical techniques. Once limited by fundamental understanding and practices, this field has developed into a sophisticated discipline that addresses complex spinal conditions with increasing precision and efficacy. The early foundations of spine surgery date back to ancient civilizations, where efforts to treat spinal disorders were largely experimental and based on limited anatomical knowledge [1,2]. Over centuries, the field has progressed from basic manipulations to more structured, evidence-based approaches [3,4]. A significant milestone occurred in the late 19th century when Dr. Victor Alexander Haden Horsley performed one of the first recorded spine surgeries, marking the beginning of modern spinal surgery with the introduction of laminectomy procedures [5].

Spine surgery and the approach to diagnosing spinal disorders have transformed in recent years and enhanced a more nuanced understanding of spine pathology. A significant change has occurred in recent decades, from reliance on clinical assessments and fundamental X-rays to incorporating advanced imaging technologies [3,4]. The introduction of computed tomography (CT) and magnetic resonance imaging (MRI) revolutionized the ability to visualize the complexity of the spine, enabling detailed preoperative planning and significantly improving diagnostic accuracy [6,7].

This review aims to investigate the paradigm shift in the diagnosis of spinal disorders, tracing the historical evolution while highlighting the modern innovations that have transformed the field. We will explore the most significant innovations in spine surgery diagnostics and discuss their implications for clinical practice and research.

## 2. Traditional Diagnostic Methods in Spinal Surgery

Diagnosing spinal disorders has undergone a significant transformation, evolving from basic clinical assessments to sophisticated imaging technologies. Understanding traditional diagnostic methods is essential to appreciate the paradigm shift in spinal surgery diagnostics.

### 2.1. Radiographic Imaging

X-ray imaging technology emerged in the early 20th century and represented a significant advantage by allowing for the noninvasive visualization of bony structures in the spine [8].

#### 2.1.1. Conventional X-Ray

Conventional X-ray is a well-established method for examining bony structures, spine alignment, fractures, and degenerative changes like osteophytes or disk space narrowing [9].

#### 2.1.2. Functional X-Rays

**Figure 1:**
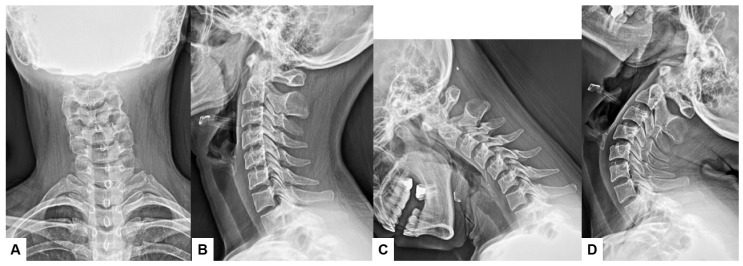
Thirty-four-year-old F, cervical spondylosis, (**A**) anteroposterior cervical conventional X-ray, (**B**) lateral conventional X-ray, (**C**) lateral flexion X-ray, (**D**) lateral extension X-ray. Cervical motion is preserved, and there is no instability.

Functional X-rays, which often involve flexion–extension, lateral bending, or other stress maneuvers, provide superior information for determining subtle displacements or intervertebral motion when compared to conventional static X-rays. Therefore, spinal disorders such as spondylolisthesis, segmental instability, or other degenerative changes can be evaluated with dynamic insights [10,11] (Figure 1C,D). Those imaging modalities are widely available in any healthcare setting and relatively inexpensive compared to advanced imaging technologies such as CT and MRI [12].

#### 2.1.3. Myelography

**Figure 2:**
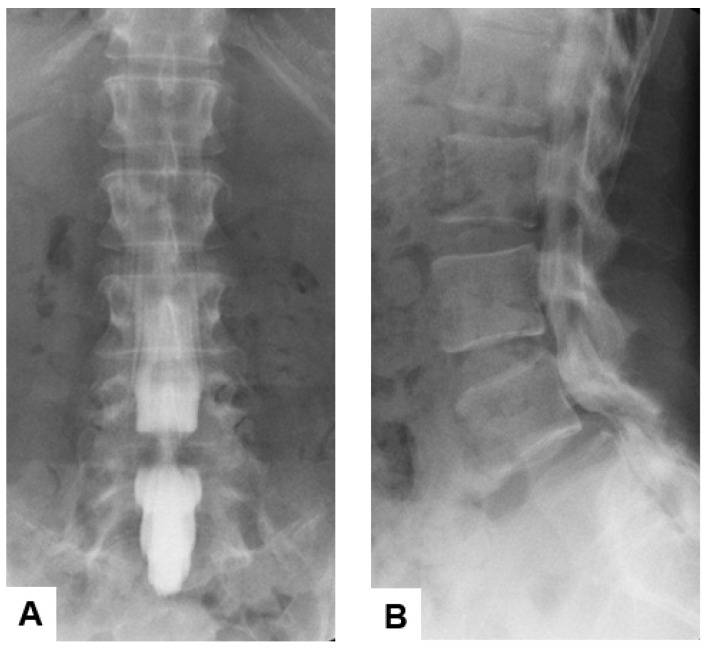
Sixty-five-year-old M, L4 lumbar degenerative spondylolisthesis, (**A**) anteroposterior lumbar myelography, (**B**) lateral lumbar myelography. The filling defect is shown at L4/5.

This radiological examination evolved in the early 20th century to enhance visualization of the spinal cord, nerve roots, and surrounding structures by injecting a radiopaque contrast agent into the subarachnoid space, followed by X-ray imaging [13]. The shape and contour of the spinal canal can be assessed by this technique, which reveals the compression or displacement of neural elements [14]. Myelography was particularly useful for diagnosing herniated disks, spinal stenosis, and tumors affecting the spinal cord or nerve roots before advanced imaging technologies [15] (Figure 2). The invasive nature and inherent risks of infection, headache, and intracranial hemorrhage due to lumbar puncture make this technique less preferable [16,17,18].

#### 2.1.4. Computed Tomography (CT)

CT has emerged as a cornerstone imaging modality for spine surgery, which provides high spatial resolution and 3D reconstruction. The multiplanar reconstruction and 3D rendering of bony structures enhance spine surgeons’ capability to assess complex deformities, detect fractures, and optimize and plan implant placement or corrective osteotomies. Moreover, the high spatial resolution allows spine surgeons to examine cortical and trabecular bones in detail, which is crucial for identifying subtle bony abnormalities [19].

### 2.2. Limitations of Traditional Radiographic Methods

Traditional radiographic methods are still the first line in the diagnostic approach of spinal disorders due to their low cost and accessibility. However, emerging advancements in imaging technology made their utilization supplementary due to limitations. The primary limitation is the lack of soft tissue visualization, where the detailed interpretation of soft tissues such as disks, ligaments, or the spinal cord is essential for any diagnosis and treatment plan of spinal disorders [20,21,22]. The secondary limitation includes radiation exposure, which necessitates judicious use, especially for the younger population, to reduce cumulative lifetime exposure [23,24,25]. Variability in patient positioning and effort during functional X-rays is another limitation, leading to diagnostic uncertainty with measurement inaccuracy. The inherent limitations of traditional methods have led technology providers to constant development.

### 2.3. Conclusion of Traditional Methods

The traditional diagnostic methods laid the groundwork for understanding spinal pathology and informed early surgical interventions. However, they also highlighted the need for more precise and less invasive diagnostic tools, paving the way for the advancements in imaging technology that followed. As the field progressed, the limitations of these traditional methods spurred innovation, leading to the development of computed tomography (CT) and magnetic resonance imaging (MRI), which have since transformed spinal surgery diagnostics.

## 3. Advances in Diagnostic Tools

The advent of advanced imaging technologies has marked a significant paradigm shift in diagnosing and managing spinal disorders, providing unprecedented insights into spinal anatomy and pathology.

### 3.1. Magnetic Resonance Imaging (MRI)

**Figure 3:**
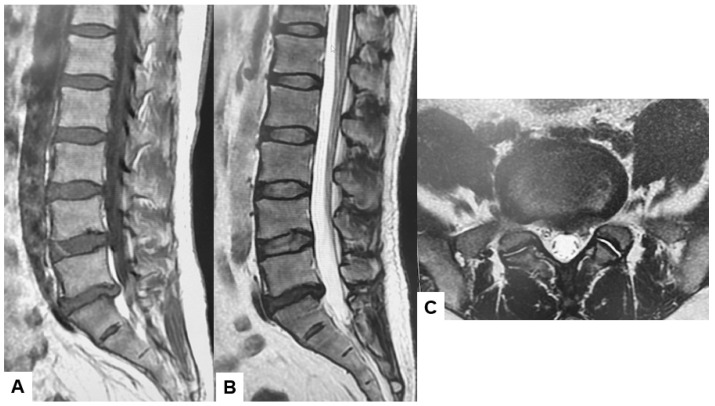
Forty-two-year-old M, left L5/S lumbar disk herniation, (**A**) T1-weighted midsagittal image, (**B**) T2-weighted midsagittal image, (**C**) T2-weighted axial image at L5/S1. Lumbar disk herniation (median and paramedian) is indicated at left L5/S1.

A major diagnostic shift occurred in the last quarter of the 20th century through the utilization of MRI [26]. This diagnostic tool revolutionized spine diagnostics by providing the high-resolution visualization of soft tissue structures, including the intervertebral disks, spinal cord, and nerves [27]. MRI utilizes the magnetic properties of protons within human tissue. When placed in a strong magnetic field, the radiofrequency pulse can align and manipulate these protons. Protons release energy captured as signals, which are then mathematically reconstructed by a computer to form detailed images. Body tissue composition, such as differences in water, fat, and protein content, contributes to the contrast seen in MRI sequences such as T1-weighted, T2-weighted, and STIR (Short Tau Inversion Recovery). This contrast allows spine surgeons to differentiate among intervertebral disks, muscles, ligaments, the spinal cord, and pathological lesions with remarkable clarity [28,29,30]. This capability is crucial for accurately diagnosing spinal pathologies. Therefore, MRI is the gold standard for spinal pathologies involving soft tissues (Figure 3).

The broad-spectrum clinical application of MRI includes degenerative spinal pathologies, spinal trauma, tumors, and infections. MRI provides superior imaging for ligamentous injuries such as ligament tears compared to CT. Moreover, spinal cord contusion, edema, and epidural hematoma can be detected in the early phases of spine trauma, which is crucial for ensuring positive spinal trauma outcomes [31,32,33]. Furthermore, contrast-enhanced MRI plays a significant role in diagnosing spinal tumors and infections, which is currently the first-line diagnostic tool [34,35,36,37].

The superior diagnostic capabilities and the ability to assist surgeons in performing meticulous preoperative planning make MRI a widely used diagnostic tool for spinal disorders [38,39,40].

### 3.2. Positron Emission Tomography (PET)

**Figure 4:**
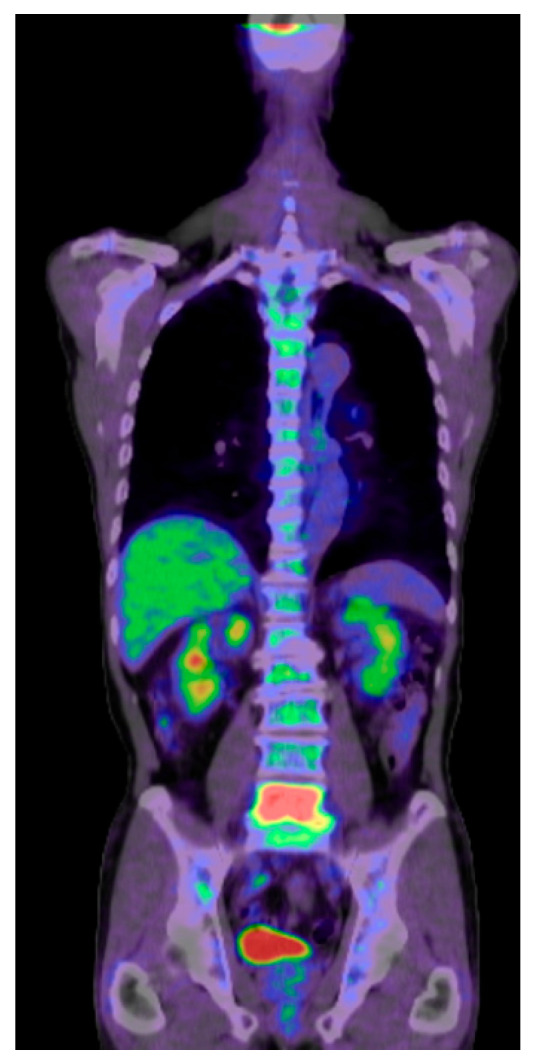
Seventy-two-year-old M, metastatic spinal tumor from maxilla cancer, PET CT, maxilla, and L5 are high uptake.

Positron Emission Tomography (PET) provides insights into metabolic processes by detecting the uptake of radiolabeled tracers in the anatomical regions where there is high cellular activity [41]. This nuclear medicine technique utilizes the functional data of PET with CT data, in which inflammatory lesions, infections, and tumors can be detected and assessed [42,43,44] (Figure 4). 18F-fluorodeoxyglucose (FDG) is the most widely used tracer, an analog of glucose. Malignant processes have a high glycolytic rate. Therefore, it is typically detected by PET/CT as strong FDG uptake. The other tracers, such as 11C-methionine, 68Ga-DOTATATE, or 18F-fluoride, are also utilized for specific tumor types or bone metabolism studies. However, no consensus exists on their usage in spine pathology [45,46].

Tumor diagnosis and staging is one of the most commonly used areas of PET/CT. This technique provides superior data to detect occult changes that are not detectable with MRI or CT [47]. Moreover, this technique is also widely used in infectious or inflammatory processes of the spine, in which FDG uptake is high [48]. Dias et al. reported that out of 4463 lesions, diffusion-weighted (DW) MRI had a pooled sensitivity and specificity of 83% and 91%, respectively, compared with 78% and 81% for LPET/CT [49].

### 3.3. CT/MRI Fusion Image

**Figure 5:**
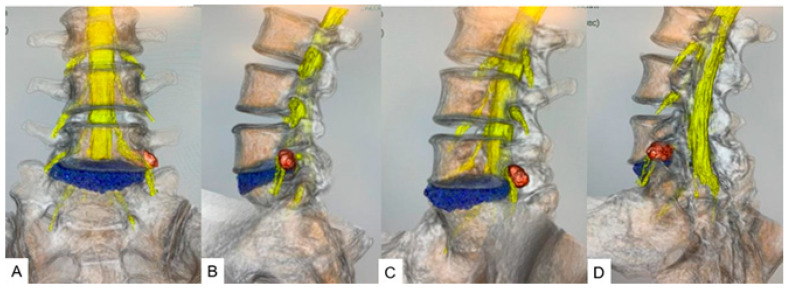
A 46-year-old male, left L5/S1 foraminal/extraforaminal disk herniation. (**A**) Anterior view. (**B**) Lateral view. (**C**) Anterior oblique view. (**D**) Posterior oblique view. Reprinted with permission from Ref. [50].

CT/MRI fusion imaging integrates the high bony resolution of CT with the superior soft tissue contrast of MRI. This technique provides comprehensive images for diagnostic and intraoperative guidance for evaluating disk herniation and vascular anatomy with bony structures [50,51]. Computer-generated three-dimensional (3D) fused models facilitate immersive preoperative planning (Figure 5).

### 3.4. Functional Magnetic Resonance Imaging (fMRI) (Figure 6)

fMRI has revolutionized the investigation of brain activity. However, the utilization of fMRI in spinal disorders is relatively limited by its technical complexity, motion, and physiological artifacts, such as the close relation of the spinal column with major blood vessels and lungs [52].

**Figure 6:**
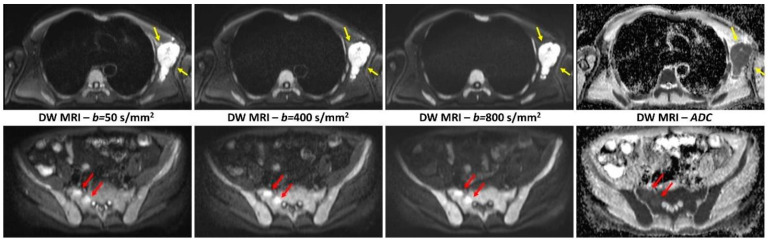
Transverse diffusion-weighted images acquired with three different *b* values (from left to right: 50 s/mm^2^, 400 s/mm^2^, and 800 s/mm^2^) and the corresponding ADC images in a 76-year-old man with bone marrow focal lesions in the pelvis (red arrows) and left axillary extramedullary disease (yellow arrows). Bone marrow diffuse involvement appears as diffuse high-signal-intensity areas on both low- and high-*b*-value diffusion-weighted images, with increased ADC values (mean 0.420 × 10^−3^ mm^2^/s). Both signal intensity on high-*b*-value diffusion-weighted images and ADC values (0.620 × 10^−3^ mm^2^/s in one pelvic lesion and 0.710 × 10^−3^ mm^2^/s in the axillary lesion) are highest in the focal lesions [57]. Reprinted with permission from Ref. [57].

The BOLD signals are utilized in traditional fMRI, which detects changes in the ratio of oxygenated to deoxygenated hemoglobin [53]. Activity in the spinal cord neurons triggers increased oxygen delivery by increasing blood flow, which alters the local magnetic resonance signals. This physical principle is well established in the brain. However, the spinal cord’s smaller vascular network and unique biomechanical environment necessitate specialized sequences and higher field strengths to detect minute signal changes [54]. Therefore, the utilization of this imaging technology is currently relatively limited. Clinical applications of this advanced technology enhance spine surgeons’ understanding of intramedullary tumors and define the functional areas within or adjacent to the tumor to preserve critical tracts during surgical procedures [55]. The literature also presents fMRI as a tool to localize functional deficits in cervical myelopathy, in which conventional MRI can only detect cord compression, whether the cord is functionally compromised or not [56] (Figure 6).

### 3.5. Upright and Kinetic MRI (Figure 7)

**Figure 7:**
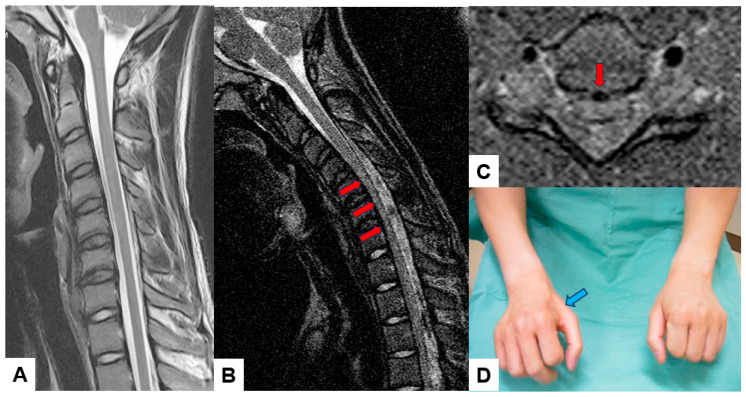
Sixteen-year-old male, Hirayama disease, (**A**) cervical midsagittal T2-weighted MRI, (**B**) cervical midsagittal MRI in flexion position, (**C**) cervical axial T2-weighted MRI at C5/6, (**D**) bilateral hands of the patients. Red arrows indicate spinal cord compression in the flexion position. A blue arrow shows muscle atrophy in his right hand.

Traditional supine MRI remains the gold standard for evaluating spinal soft tissue, but weight-bearing or dynamic conditions are essential for spine pathologies. Therefore, this technique is developed to diagnose the full spectrum of spinal disorders, including inherent instabilities or positional nerve root compressions (Figure 7).

A vertically oriented magnet allows the patient to be scanned, standing, or seated. In contrast, kinetic MRI utilizes the same upright MRI machine with multiple dynamic or functional positions [58,59].

The main limitations of this imaging modality are the lower magnetic field strengths (0.5T–0.6T), which compromise the image resolution, longer exam duration, and motion artifacts [60].

### 3.6. Magnetic Resonance Spectroscopy (MRS)

Magnetic resonance spectroscopy (MRS) is an advanced MRI-based technique that provides a noninvasive measurement of the chemical composition of tissues. This technique has primarily been more widely used in brain imaging. However, emerging research suggests that it can provide unique information about spinal pathologies, such as detecting biochemical markers linked to tumors, infections, degenerative disk disease, and postoperative changes [61]. MRS measures the relative concentrations of metabolites based on their unique resonance frequencies in a strong magnetic field. Common metabolites in spinal tissue include the following [62]:

Choline: Often elevated in highly proliferative tissues (e.g., tumors, inflammation) due to increased cell membrane turnover. Creatine (Cr): As an energy reserve marker, it is relatively stable in most tissues; it is often used as a reference for comparing other metabolite levels. N-Acetyl Aspartate (NAA): Typically abundant in healthy neural tissue; decreases can indicate neuronal or axonal damage. Lactate (Lac): Associated with anaerobic metabolism, often elevated in abscesses, necrotic tumors, or hypoxic conditions. Lipids: Lipid peaks can be elevated in necrotic or degenerative processes, as well as in certain high-grade tumors.

Magnetic resonance spectroscopy represents a promising adjunct to conventional MRI in spine surgery. It offers biochemical insights into spinal tissues but remains costly and technically challenging.

### 3.7. Diffusion-Weighted Imaging (DWI) and Diffusion Tensor Imaging (DTI)

Diffusion-weighted imaging (DWI) and diffusion tensor imaging (DTI) are considered advanced magnetic resonance imaging (MRI) techniques that provide information about the microstructure of tissue by measuring the diffusion of water molecules. This technique is primarily used for brain imaging, similar to other advanced MRI modalities. However, both DWI and DTI have seen increasing use in the spine. This technique allows us to examine the microstructural changes in the spinal cord, nerve roots, and intervertebral disks, even when conventional imaging appears normal. DWI primarily measures the degree of water diffusion (i.e., how freely water molecules move in tissue). DTI extends this by mapping the orientation of diffusion in three-dimensional space, providing metrics such as fractional anisotropy (FA) and mean diffusivity (MD) that yield insights into axonal integrity, fiber density, and demyelination [63].

DWI and DTI hold significant promise for advancing spine surgery by offering the enhanced visualization of microstructural changes in the spinal cord, nerve roots, and intervertebral disks, thus enhancing the diagnosis of early cervical myelopathy, spinal tumor differentiations, and evaluation of response to treatment.

## 4. Emerging Technologies

### 4.1. Artificial Intelligence and Machine Learning

**Figure 8:**
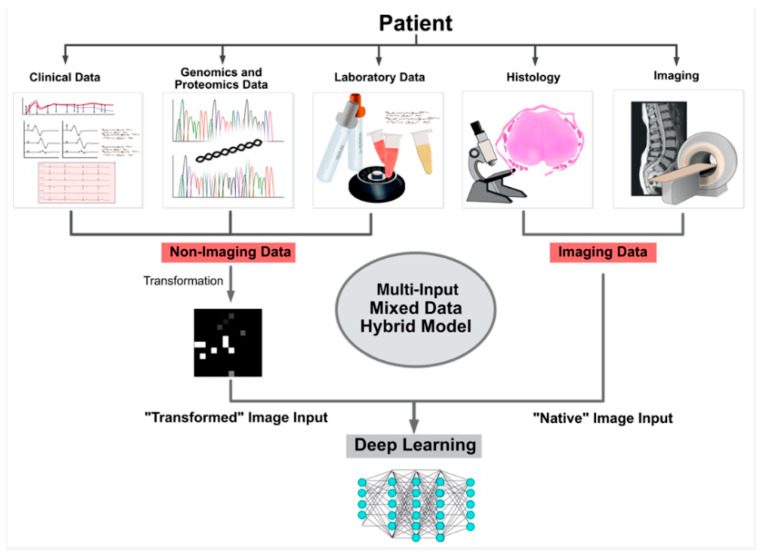
Illustration of multi-input mixed data model application in clinics [67]. Reprinted with permission from Ref. [67].

Integrating artificial intelligence (AI) and machine learning (ML) into spinal diagnostics represents a transformative advancement, offering both efficiency and precision (Figure 8). AI has some limitations, such as data bias, interpretability challenges, and regulatory hurdles.

Pattern Recognition: AI algorithms excel at pattern recognition and can analyze imaging data to detect subtle abnormalities the human eye may miss [64]. This enhances diagnostic accuracy and consistency.

Predictive Analytics: Machine learning models can predict disease progression and treatment outcomes by analyzing large datasets, aiding in personalized treatment planning [65].

Automated Systems: AI-driven systems can automate routine tasks, reducing the time radiologists and surgeons spend on these activities [66].

### 4.2. 3D Modeling, Augmented Reality (AR), and Virtual Reality (VR)

**Figure 9:**
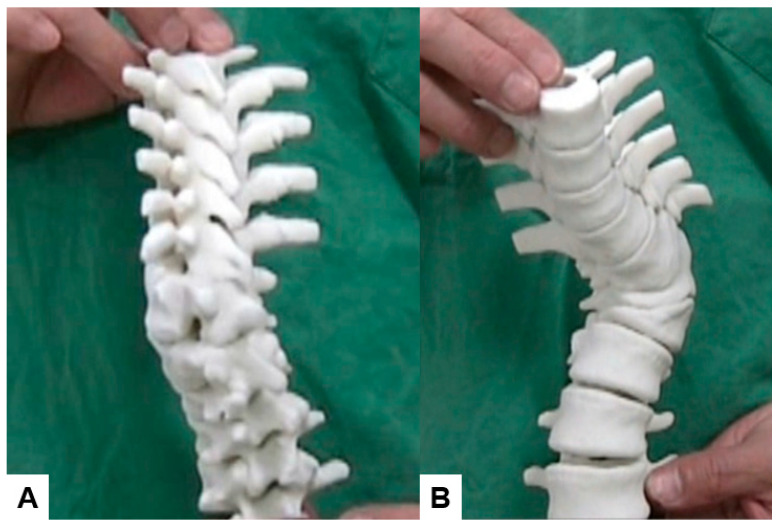
Three-dimensional model of congenital kyphoscoliosis. (**A**) oblique view; (**B**) anterior view. This model is helpful for preoperative surgical planning.

Innovations in three-dimensional (3D) rendering and virtual reality enable spine surgeons to simulate and plan procedures in an immersive environment. By converting CT or MRI scans into detailed anatomical reconstructions, surgeons can better visualize complex pathologies, practice surgical approaches, and anticipate challenges. Preliminary studies suggest that 3D models can significantly improve preoperative planning, reduce operative times, and minimize complications [68,69] (Figure 9).

Augmented reality (AR) and virtual reality (VR) are expected to benefit spinal surgery. VR and AR are important navigation tools in the operating room. Furthermore, AR and VR have already shown benefits in several areas of healthcare, such as robotic surgery [70].

### 4.3. Wearable Technology and Remote Monitoring

**Figure 10:**
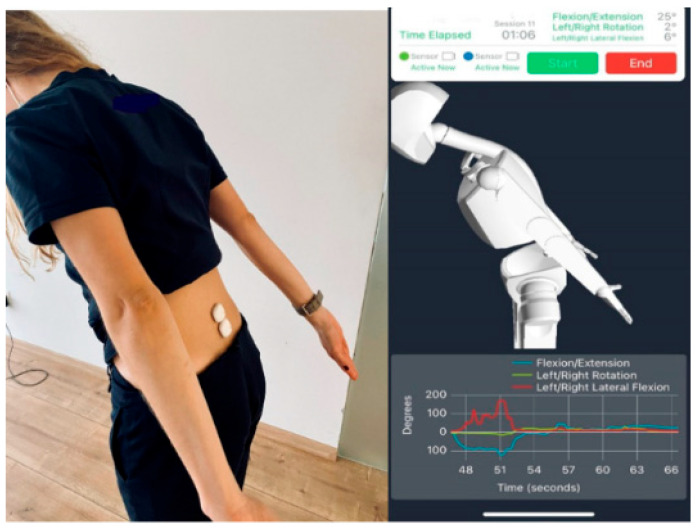
Wearable sensors. Wearable sensors’ application at the lumbar spine. Software reproduction—kinematic data recording [73]. Reprinted with permission from Ref. [73].

Wearable devices equipped with accelerometers and gyroscopes track patient mobility and posture in real-world settings, providing continuous data that can complement imaging findings. Recently, there has been an emphasis on using wearable devices to measure physical activity and limb and spine function [71]. One good example is wearable sensors for lumbar kinematic measurements (Figure 10). Telemedicine platforms further allow clinicians to remotely monitor patient-reported outcomes, rehabilitation progress, and potential complications. This continuous feedback loop fosters proactive adjustments in the diagnostic and therapeutic plan [71,72].

### 4.4. Genetic Testing and Biomarkers

**Figure 11:**
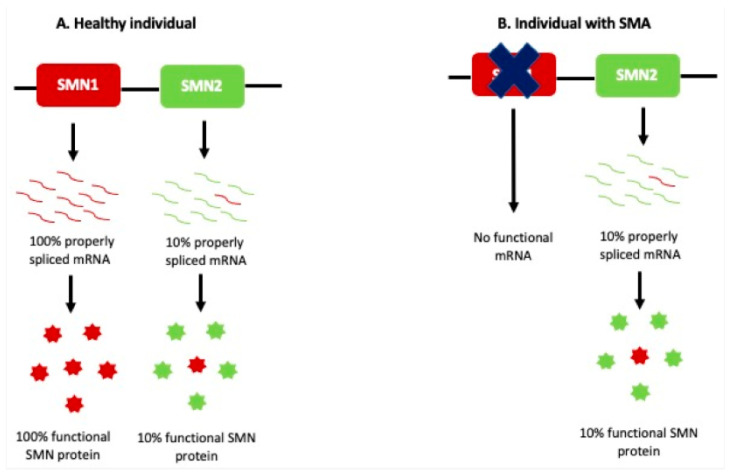
Etiology of SMA. (**A**) *SMN1* can produce 100% properly spliced mRNA, which is translated to functional SMN protein in healthy individuals. *SMN2* can produce only 10% functional mRNA transcripts, while the remaining 90% *SMN2* transcripts lack exon 7 and are rapidly degraded. (**B**) Patients with SMA do not have *SMN1* and rely on the 10% SMN protein produced by *SMN2*. This cannot compensate for the loss of *SMN1* [74].

Research into the genetic and molecular underpinnings of spinal diseases is revealing novel biomarkers that may predict disease progression and therapeutic responses. For instance, certain collagen and matrix metalloproteinase (MMP) gene polymorphisms correlate with an increased risk of early disk degeneration. When used alongside imaging, biomarker profiling promises a more holistic understanding of each patient’s condition and tailors a more individualized treatment approach [74,75]. For the diagnosis of spinal muscular atrophy (SMN), biomarkers of SMA protein, the number of gems, and deletion of the SMN1 gene are applied (Figure 11) [76].

Table 1 summarizes the advantages and disadvantages of all spinal modalities.

## 5. Challenges and Limitations in Current Paradigms

While the paradigm shift in spinal surgery has brought about significant advancements in diagnosis and treatment, it has also introduced a range of challenges and limitations. The cruD model of congenital kyphoscoliosis impacts the implementation and broader adoption of new technologies and practices in the field. Key areas of concern include financial and resource limitations, training and skill acquisition for new technologies, and ethical and regulatory challenges.

Emerging technologies, data-driven decision-making, and innovative educational tools are set to shape the future of spinal surgery. Integrating AI and robotics, along with advancements in data analytics and virtual reality, promises to enhance spinal procedures’ precision, safety, and efficacy. As these technologies continue to develop, they will play a critical role in transforming spinal surgery, offering new opportunities for improving patient outcomes and expanding the capabilities of surgical education.

## 6. Discussion

Spinal surgery has undergone a remarkable transformation, driven by advancements in diagnostic tools, surgical techniques, and patient-centered care, especially in the last three decades. Rapid development in computer technology has integrated with the diagnostic field, constituting an inevitable diagnostic paradigm shift. Innovations in imaging technology, computer-assisted analysis, and trends in personalized patient care drive this. Conventional X-ray is still widely used worldwide as a first-line imaging modality due to its accessibility and low cost. However, treatment decisions and planning are usually executed after a CT or MRI scan. Those modalities are superior for assessing bone and soft tissue in a detailed 3D manner. MRI represents irrefutably the major diagnostic paradigm shift in the 20th century for spinal pathologies, for which, in 2003, MRI developers were awarded the Nobel Prize (Poul Christian Lauterbur, Peter Mansfield).

Approximately 5 billion diagnostic examinations are estimated to be performed worldwide annually [75]. Studies have shown a constantly increasing trend in MRI scans [76,77]. This is probably an indicator of more advanced imaging technologies becoming available worldwide. Thus, the diagnostic capability of detecting spinal disorders inevitably evolves each year. However, this increase in the utilization of advanced imaging technologies brings about the issue of the financial burden of diagnostic technologies in healthcare systems. Meticulous clinical examination is essential for spinal disorders to utilize the diagnostic methods judiciously. This approach is a key element of any disease diagnostic protocol, as healthcare resources are scarce.

Advancements in computer technology over the last three decades led to the development of specific high-end MRI-based diagnostic methods. Functional MRI has transformed our comprehension of brain activity, yet its utilization in the spinal cord is relatively limited. Stroman et al. [34] demonstrated that people with complete SCI still showed BOLD signal changes with fMRI in the spinal cord when applying thermal stimuli. This indicates that fMRI will undoubtedly be used in increasing trend SCI research. A recent systematic review concluded that DTI has promising results as a clinical tool for diagnosis, prognosis, recovery, and treatment efficacy in SCI. However, the limitations of this technique impede its transition beyond research into clinical practice [78]. However, increased high-sensitivity imaging such as XX may lead to unnecessary interventions. Surgeons should evaluate patients’ symptoms related to those images.

The spine is the key element of our axial skeleton; therefore, load bearing plays a vital role in detecting inherent instabilities. Kinetic MRI has emerged as a solution to detect positional changes; however, authors believe that the information obtained from this technique does not justify its cost. Moreover, conventional dynamic X-rays are still valid for detecting inherent instabilities with lower costs.

Emerging technologies and data-driven decision-making are set to shape the future of spinal surgery. A recent systematic review by Han et al. [79] showed that integrating AI and advancements in data analytics and virtual reality promises to enhance spinal procedures’ precision, safety, and efficacy. As these technologies continue to develop, they will play a critical role in transforming spinal surgery, offering new opportunities for improving patient outcomes and expanding the capabilities of surgical education. Combining all emerging technologies will provide sophisticated and tailored solutions for patients. This paradigm shift emphasizes the importance of continuous innovation and adaptability in improving the diagnosis and treatment of spinal disorders.

While promising, the paradigm shift in spinal surgery is accompanied by several challenges and limitations that must be addressed to maximize the benefits of new technologies. Financial constraints, the need for specialized training, and ethical and regulatory considerations are pivotal in shaping the landscape of modern spinal surgery. Addressing these issues through strategic planning, investment in education, and collaboration between stakeholders will be essential to overcome barriers and ensure that advancements in spinal surgery are accessible and beneficial to a broader patient population.

Diagnostic technologies strongly support physicians and enhance clinical practice from screening to prognosis, by detecting diseases early, and by monitoring them with increasing accuracy. Rapid development in computer technology inevitably induces a diagnostic paradigm shift in spine surgery. This paradigm shift emphasizes the importance of continuous innovation and adaptability in improving the diagnosis and treatment of spinal disorders.

## Figures and Tables

**Table 1:** Advantages and disadvantages of all modalities.

**Modality**	**Advantage**	**Disadvantage**
CT	Bony structure	Radiation risk
MRI	Soft tissue	Artifact
PET	Detect malignancy	Availability
CT/MRI fusion image	Bone and soft tissue	Availability
fMRI	Functional	Poor image quality
Upright and kinematic MRI	Functional	Expensive
MRS	Chemical composition	Very expensive
DWI and DTI	Microstructure	Poor image quality
Artificial intelligence	Limitless possibility	Interpretability challenges
3D model, AR, VR	Surgical planning	Cost
Wearable technology	Continuous data	Data security
Genetic testing, biomarkers	Early detection	Psychological stress
